# A Detailed Epidemiological and Clinical Description of 6 Human Cases of Avian-Origin Influenza A (H7N9) Virus Infection in Shanghai

**DOI:** 10.1371/journal.pone.0077651

**Published:** 2013-10-15

**Authors:** Jindong Shi, Juan Xie, Zebao He, Yunwen Hu, Yanchao He, Qihui Huang, Beizheng Leng, Wei He, Ying Sheng, Fangming Li, Yuanlin Song, Chunxue Bai, Yong Gu, Zhijun Jie

**Affiliations:** 1 The Fifth People's Hospital of Shanghai, Fudan University, Shanghai, China; 2 Department of Pathogen Diagnosis and Biosafety, Shanghai Public Health Clinical Center, Shanghai, China; 3 Department of Pulmonary Medicine, Zhongshan Hospital, Fudan University, Shanghai, China; University of Hong Kong, Hong Kong

## Abstract

**Background:**

The world’s first reported patient infected with avian influenza H7N9 was treated at the Fifth People’s Hospital of Shanghai. Shortly thereafter, several other cases emerged in the local area. Here, we describe the detailed epidemiological and clinical data of 6 cases of avian influenza H7N9.

**Methods and Findings:**

We analyzed the epidemiologic and clinical data from clustered patients infected with H7N9 in the Minhang District of Shanghai during a 2-week period. Of the 6 patients, 2 were from a single family. In addition, 3 patients had a history of contact with poultry; however, all 6 patients lived in the proximity of 2 food markets where the H7N9 virus was detected in chickens and pigeons. The main symptoms were fever, cough, and hemoptysis. At onset, a decreased lymphocyte count and elevated creatine kinase, lactate dehydrogenase, procalcitonin, and C-reactive protein levels were observed. As the disease progressed, most patients developed dyspnea and hypoxemia. Imaging studies revealed lung consolidation and multiple ground-glass opacities in the early stage, rapidly extending bilaterally. All patients were treated with oseltamivir tablets beginning on days 3–8 after onset. The main complications were as follows: acute respiratory distress syndrome (ARDS; 83.3%), secondary bacterial infection (66.7%), pleural effusion (50%), left ventricular failure (33.3%), neuropsychiatric symptoms (33.3%), and rhabdomyolysis (16.7%). Of the 6 patients, 4 died of ARDS, with 2 patients recovering from the infection.

**Conclusions:**

An outbreak of H7N9 infection occurred in the Minhang District of Shanghai that easily progressed to acute respiratory distress syndrome. Two cases showed family aggregation, which led us to identify the H7N9 virus and indicated that human transmission may be involved in the spread of this infection.

## Introduction

Avian influenza is an infectious poultry disease caused by avian influenza A viruses, which are classified as low pathogenic avian influenza A (LPAI) and high pathogenic avian influenza A (HPAI) viruses. LPAI viruses rarely cause severe avian diseases, whereas HPAI viruses can cause deadly infectious diseases in birds, spreads rapidly, and can infect humans. Almost all HPAI viruses belong to the H5, H7, and H9 subtypes. The main subtypes of avian influenza viruses known to infect humans are H7, H5, H9, and H10 and N1, N2, N3, and N7. There have been over 100 documented cases of humans infected with H7 viruses from poultry or wildfowl mainly in the United States, United Kingdom, Italy, the Netherlands, Canada, and other European countries [1-6], while N9-subtype viruses had never been reported in humans until recently. Most human infections with avian influenza A (H7) viruses exhibit mild symptoms or present as latent infections. Most cases result in conjunctivitis and symptoms of respiratory tract infection that are mild to moderate in severity, with only one fatal case reported in a patient with H7N7 virus infection [7]. From 1999 to 2003, Puzelli found that 7 of 185 poultry workers (3.8%) were positive for specific antibodies to avian influenza A H7 viruses, of which only 1 case resulted in conjunctivitis, whereas the remaining 6 cases exhibited no clinical symptoms [4]. By May 28, 2013, 123 patients were diagnosed with laboratory-confirmed H7N9 virus infections and admitted to the hospital, and 37 (30%) patients died of the infection; this outcome is inconsistent with those of previous reports on avian influenza A H7 virus infections [8].

In February 2013, the first ever case of human infection with H7N9 was encountered at the respiratory department of the Fifth People’s Hospital of Shanghai [9]. During the following 2-week period, the hospital received another 5 patients with H7N9 infection. All cases were localized to the Minhang District of Shanghai, and the disease onset in all cases occurred within a 2-week period. Of the 6 patients, 2 emerged from a single family, suggesting that aggregation exists to some extent. In addition, H7N9 avian influenza and H5N1, both of which are fatal, show similar clinical symptoms and disease progression [10,11]. Therefore, early diagnosis and treatment, with an improved cure rate are important to control these infections [11]. Here, we summarize the findings of 6 cases of pneumonia caused by H7N9 infection.

## Methods

### Ethics statement

This work was approved by the Human Research Committee at the Fifth people`s hospital of Shanghai and Shanghai Public Health Clinical Center, and written informed consent was obtained from the patients or their families.

### Subjects

All confirmed cases of H7N9 referred to the Fifth People’s Hospital of Shanghai during the period from February to March 2013 were enrolled in this study. H7N9 infection was confirmed by the Public Health Clinical Center Affiliated with Fudan University as well as the Chinese Center for Disease Control and Prevention.

### Collection of clinical data and laboratory specimens

General patient information, clinical signs and symptoms, laboratory test results, chest radiographic findings, and treatment were recorded. Information regarding the patients’ recent activities and environment was also analyzed. Clinical signs and laboratory testing results refer to testing that was performed before the patients were hospitalized or within 2 days of hospitalization.

### Viral detection

Throat-swab, sputum, and blood specimens obtained from all patients with suspected H7N9 infection were sent to the Shanghai Public Health Clinical Center. A specific reverse transcriptase-polymerase chain reaction (RT-PCR) assay for avian influenza H7N9 was used to detect the virus in throat-swab and sputum specimens. In addition, H7N9 virus was also isolated from throat-swab and sputum specimens. Serum samples from the 2 surviving patients obtained at both the acute phase and the recovery stage were tested for specific antibodies to H7N9 virus.

## Results

### Demographic characteristics

All 6 patents were male, Han nationality, aged from 27 to 87 years old, 4 were retired individuals, 1 in-service worker, and 1 pork peddler. Four patients had history of tobacco, 1 had history of drinking, and 5 had at least 1 of the following underlying diseases: chronic obstructive pulmonary diseases, hypertension, dextrocardia, diabetes, coronary heart disease, hepatitis B, and/or gastric ulcer. (Table 1)

**Table 1:** Demographic characteristics of 6 H7N9 Cases.

Variable	Case 1^a^	Case 2	Case 3	Case 4	Case 5	Case 6
Age (y)	87	69	74	27	41	63
Sex	Male	Male	Male	Male	Male	Male
Nationality	Han	Han	Han	Han	Han	Han
Occupation	Retired	Retired	Retired	Butcher^b^	Worker	Retired
Place of Residence	Minhang District of Shanghai	Minhang District of Shanghai	Minhang District of Shanghai	Jiangsu province	Minhang District of Shanghai	Minhang District of Shanghai
Smoking index (pack-years)	>30	No	30	No	4	15
History of alcohol intake	No	No	No	No	No	Alcoholism
Underlying conditions	COPD, hypertension, dextrocardia	Hypertension	Hypertension, diabetes mellitus, coronary heart disease	Hepatitis B^c^	No	COPD, hypertension, gastric ulcer

^a^ Case 1 was the father of case 2, whose other son showed similar symptoms on February 11 and subsequently died from severe pneumonia on February 28; however, H7N9 infection was not confirmed in his case.

^b^ Case 4 sold pork in the market, but had not butchered bird meat.

^c^ Case 4 was found to have a history of HBV infection after admission.

### The time course of case identification, treatment, and diagnosis

Disease onset in the 6 cases occurred within a 2-week period from 19 February and 5 March 2013. The time range from onset to hospitalization was 3–7 days (mean, 4.6 days). Cases 3 and 4 were admitted to the intensive care unit (ICU) because of disease progression on the first and second day of hospitalization, respectively. The length of hospital stay was 3–15 days (mean, 8 days). H7N9 was confirmed by RT-PCR and virus isolation in 4 cases and by elevated (4× that of normal) levels of specific antibodies to H7N9 in the acute phase and recovery stage in 2 cases. Case 2 was the son of case 1, whose other son (age, 55 years) developed severe pneumonia on February 11 and died on February 28. However, the H7N9 virus was not detected in respiratory specimens from the deceased son by RT-PCR or viral isolation. (Table 2)

**Table 2:** Date of disease onset, admission, confirmed infection, and discharge of 6 H7N9 cases.

**Variable**	**Case 1**	**Case 2**	**Case 3**	**Case 4**	**Case 5**	**Case 6**
Date of illness onset	Feb 19	Feb 19	Feb 24	Feb 27	Mar 3	Mar 5
Date of admission	Feb 25	Feb 26	Feb 27	Mar 4	Mar 6	Mar 9
Admission to ICU	No	No	Yes	Yes	No	No
Confirmed infection	Mar 30	Apr 12	Apr 13	Mar 30	Apr 13	Apr 14
Method of confirmation	Viral isolation	Virus specific antibodies (1:40)	Viral isolation	Viral isolation	Virus-specific antibodies (1:40)	Viral isolation
Length of hospital stay (d)	6	15	5	7	12	3
Family aggregation			-	-	-	-
Recent exposure to poultry	No	Suspected^a^	No	Yes	Yes	No
Date of death	Mar 4	-	Mar 3	Mar 10	-	-
Date of discharge	-	Mar 13	-	-	Mar 18	-

^a^ Case 2 had no history of direct contact with birds, but had gone to the market several times 1 week before disease onset and passed by stalls selling poultry.

### History of exposure to birds and residence state

**Figure 1:**
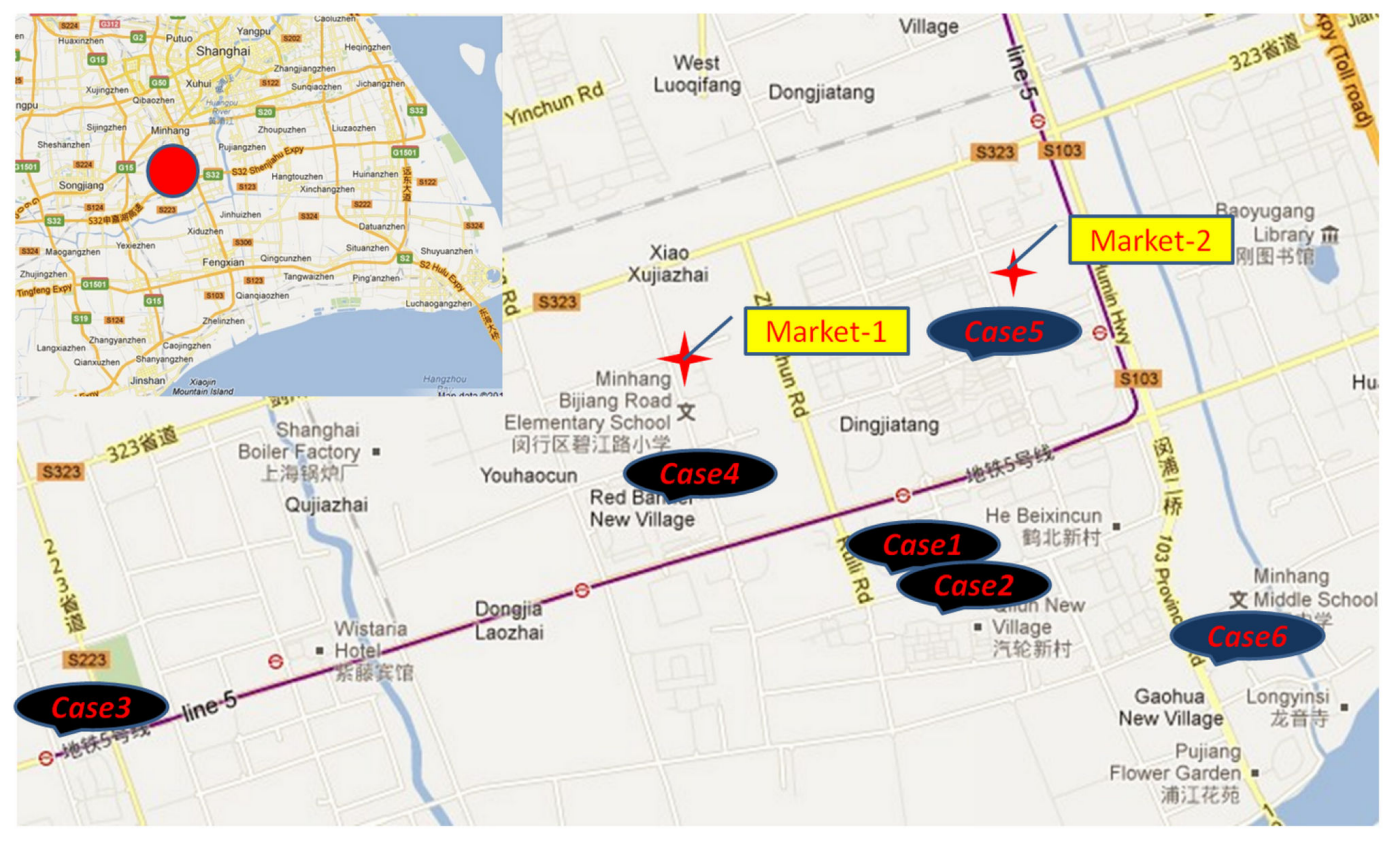
A map of patient residences and the 2 neighboring markets where H7N9 was detected in poultry.

Among the 6 patients, 5 were residents of the Minhang District of Shanghai and had not left Shanghai prior to the onset of illness. One patient, who was a pork seller at a market in the Minhang District, was originally from the Jingsu province and had been a resident of Minhang District for 9 months at the time of disease onset. All 6 patients lived in the proximity of 2 food markets where poultry were traded and H7N9 virus carrier birds had been discovered. Trading of poultry was banned in the markets on April 4. Two patients had a history of exposure to live birds and 1 had a history of suspected exposure. (Figure 1)

### Medical observation of the exposed population

There were 122 people who were in close contact with the 6 infected patients, mainly consisting of family members, relatives, and medical staff. All close contacts were kept under medical observation for 1 week, and clinical signs and symptoms similar to those of the patients were not observed.

### Signs and symptoms

All patients had fever (100%), with 5 (83.3%) patients developing high fever (temperature, 38.6–41°C) and 1 patient with chills and shivering. All patients had cough, 4 (66.7%) of whom had white sputum, 1 had yellow sputum, and 5 had hemoptysis. Five patients (83.3%) had dyspnea. One patient (16.7%) had evident chest pain. Two patients had nausea and vomiting. Patient symptoms are shown in Table 3.

**Table 3:** Signs and symptoms in 6 cases of H7N9 infection.

	**Case 1**	**Case 2**	**Case 3**	**Case 4**	**Case 5**	**Case 6**	**Percentage (%)**
Fever (°C)	40.2	40	38.6	40.2	39.9	40	100
Chills	-	-	-		-	-	16.7
Cough							100
White sputum				-		-	66.7
Yellow sputum	-	-	-		-	-	16.6
Emptysis						-	83.3
Dyspnea					-		83.3
Chest pain	-	-	-		-	-	16.7
Nausea and vomiting			-	-	-	-	33.3

### Laboratory findings

Blood cell count and biochemical test results were obtained prior to admission or within 2 days of admission. White blood cell (WBC) counts were normal or below normal levels. In particular, the lymphocyte and platelet counts were low in 4 critical patients. In addition, all 6 patients had elevated aspartate aminotransferase levels, 5 of whom had elevated creatine kinase (CK) and lactate dehydrogenase (LDH) levels; these laboratory results were missing for Case 5. The C-reactive protein (CRP) levels of all patients were low, and 5 patients had elevated β-natriuretic peptide levels, which was not tested in case 5. Blood gas analysis suggested that 5 patients had hypoxemia, and 4 patients had decreased PCO_2_ and elevated pH values as a result of hyperventilation. Laboratory findings are shown in Table 4.

**Table 4:** Laboratory findings in the early stage of H7N9 infection in 6 cases.

	**Case 1**	**Case 2**	**Case 3**	**Case 4**	**Case 5**	**Case 6**
WBC (×10^9^/L)	4.66	2.62 ↓	4.01	2.13 ↓	4.54	12.57
Lymphocytes (×10^9^/L)	0.53 ↓	0.3 ↓	0.49 ↓	0.23 ↓	0.75 ↓	0.4 ↓
Hb (g/L)	114	131	142	143	156	113
PLT (×10^9^/L)	78 ↓	92 ↓	83 ↓	32 ↓	119	430
AST (U/L)	77 ↑	80 ↑	67 ↑	58 ↑	71 ↑	56 ↑
ALT (U/L)	31	39	33	41	54	48
LDH (U/L)	1929 ↑	658 ↑	691 ↑	2327 ↑	NA	789 ↑
CK (U/L)	501↑	735 ↑	811 ↑	2932 ↑	NA	216 ↑
CK-MB (U/L)	27	23	39	47	NA	11
cTNTc (ng/mL)	0.05	0.02	0.11	0.02	NA	<0.012
BNP (ng/mL)	7480 ↑	258↑	4090 ↑	NA	NA	3000 ↑
Procalcitonin (ng/mL)	0.327↑	0.188↑	11.13↑	0.4094↑	0.196↑	0.244↑
CRP (mg/L)	114↑	41↑	10	32↑	42↑	>160↑
ESR (mm/h)	8	NA	NA	4	32↑	98↑
Urea nitrogen (mmol/L)	6.7	3.5	10.9↑	3.8	4.9	5.5
Creatinine (µmol/L)	74	74	102	72	94	57
pH	7.48↑	7.48↑	7.38	7.44	7.51↑	7.51↑
PCO_2_ (mEq/L)	29↓	35	40	38	28↓	26↓
PO_2_ (mmHg)	54↓	87	57↓	49↓	64↓	64↓
BE (mEq/L)	-0.1	3.6	2.4	2.5	1.3	-0.2

^↓^ lower than reference value

^↑^ higher than reference value

### Imaging features

**Figure 2:**
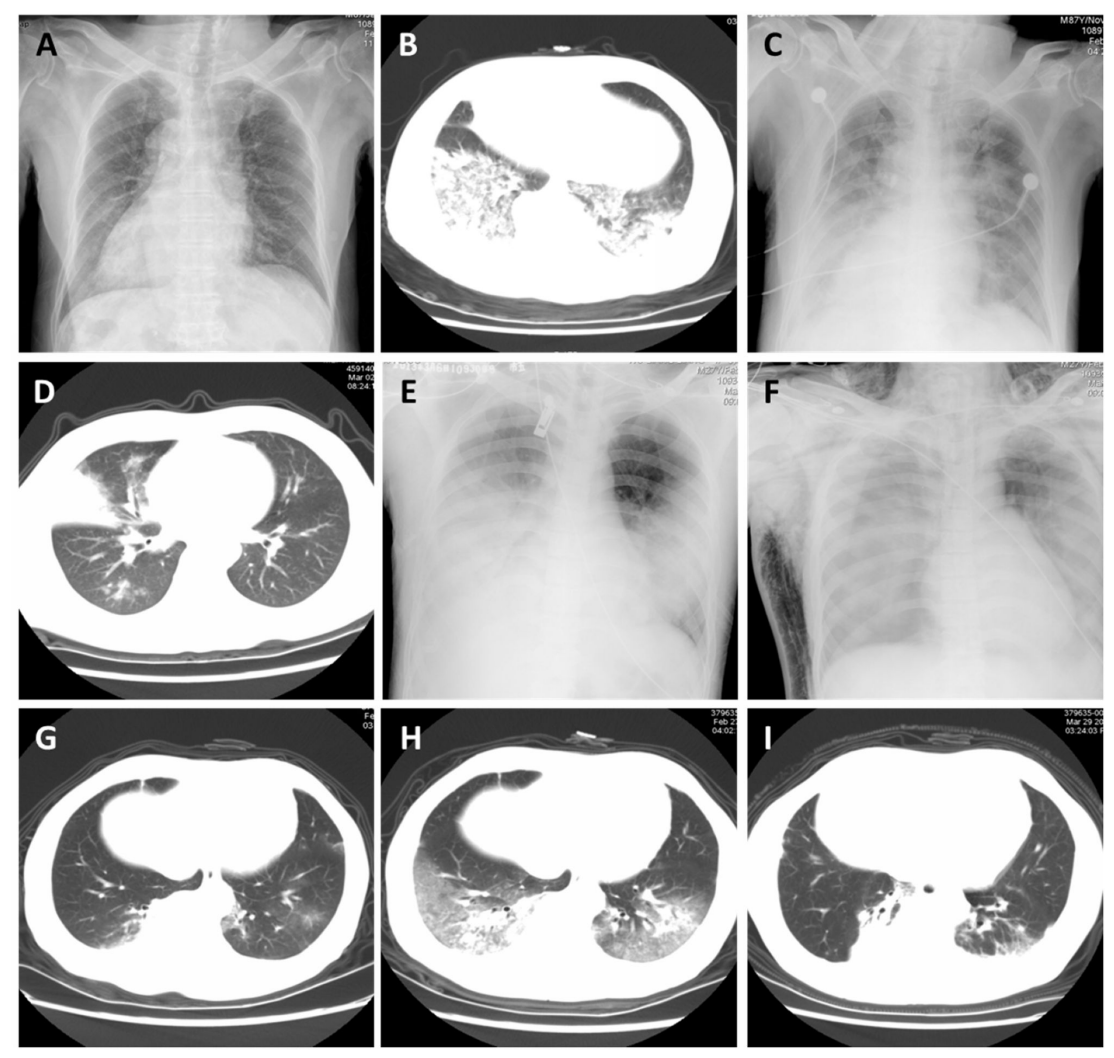
Chest radiographic findings and progression of pneumonia in 6 patients with H7N9 infection. Case 1: Panels A, B, and C; Case 4: Panels D, E, and F; Case 2: Panels G, H, and I. Early stage (panels A, D, and G): Chest computed tomography and radiograph show progressive consolidation in the lungs; Progressing stage (panels B, E, and H): Chest computed tomography and radiograph show bilateral ground-glass opacity and consolidation; Final stage (panels C, F, and I): Chest computed tomography and radiograph show diffuse lesions and bilateral involvement of the lungs (white lungs) in panels C and F, subcutaneous emphysema with pneumomediastinum in panel F, and diffuse lesions are obviously seen in panel I.

Chest radiography and lung computed tomography suggested that consolidation and multiple ground-glass opacities emerged in the early stage of disease and rapidly extended bilaterally. In 2 of 6 cases, pulmonary symptoms such as acute respiratory distress syndrome (ARDS) were detected. Furthermore, minor pleural effusion and subcutaneous and mediastinal emphysema were observed (Figure 2).

### Treatment, complications, and outcome

Oseltamivir (tablet, 75 mg bid) therapy was initiated 3–8 days after disease onset in all patients. One recovering patient was treated with a combination of oseltamivir (tablet, 75 mg bid) and amantadine (tablet, 0.2, bid). Broad-spectrum antibiotics against Gram-positive and Gram-negative bacteria and atypical pathogens, such as penicillium, carbon alkene, and fluoroquinolone, were administered to all patients. Except for the recovering patient, all patients were treated with intravenous glucocorticoid as an anti-inflammatory at a dose of 80–240 mg/d. Intravenous immunoglobulin was administered in 4 patients, and thymosin was administered in 2 patients.

In terms of complications, 4 patients developed ARDS between days 3 and 9 (mean, day 6) after disease onset. Three patients had secondary bacterial infections, 3 had pleural effusion, 2 had left heart functional failure, 2 had neuropsychiatric symptoms, and 1 had rhabdomyolysis. Among the 2 recovering patients, 1 patient had no complications, while the other developed secondary bacterial infections. (Table 5)

**Table 5:** Treatment, complications, and outcome of H7N9 infection in 6 cases.

		**Case 1**	**Case 2**	**Case 3**	**Case 4**	**Case 5**	**Case 6**
**Antibiotic**		+	+	+	+	+	+
**Antiviral**	Oseltamivir	75 mg bid d8–14^a^	75 mg bid d7–12^a^	75 mg bid d3–7^a^	75 mg bid d7–11^a^	75 mg bid d8–13^a^	75 mg bid d5–6^a^
	Amantadine	-	-	-	-	0.2 bid d8–12	-
**Anti-inflammatory**	Methylprednisolone	+	+	+	+	-	+
	Globulin	-	+	+	+	-	+
	Thymic peptide	+	+	-	-	-	+
	Albumin	+	-	-	+	-	-
**Oxygen therapy**		Mask^b^	Mechanical ventilation	Nasal catheter	Mechanical ventilation	Nasal catheter	Mechanical ventilation
**Complications**	ARDS	d9^a^	-	d3^a^	d7^a^	-	d5^a^
	Secondary Infection	+	+	-	+	-	-
	Pleural effusion	+	-	-	+	-	+
	Encephalopathy	+	-	-	-	-	+
	Left heart failure	-	-	+	-	-	+
	Rhabdomyolysis	-	-	-	+	-	-
**Outcome**		Died	Survived	Died	Died	Survived	Died

^a^ Days from disease onset

^b^ Case 1 refused intubation and mechanical ventilation

## Discussion

H7N9 is a subtype of the avian influenza A virus. Until 2013, 25 types of H7N9 viruses had been detected in birds worldwide. Previously detected H7N9 viruses only infected birds, and these infections were generally mild. However, animal experiments showed that both HPAI H7N3 and LPAI H7N9 could effectively replicate in mice without adaptation, as well as in the upper and lower respiratory tract of ferrets. HPAI H7N3 was also found to be lethal in mice and showed transmission through contact in the ferret model, whereas LPAI H7N9 could not be transmitted in the ferret model [12-15]. In March 2013, 3 cases of human H7N9 avian influenza infection were reported in Shanghai and Anhui, China. Research showed that the H7N9 virus reported at this time was a novel, reassortant influenza A virus [9]. The virus contained 8 gene segments, with only the NA gene being closely related to that of another H7N9 virus (KO14). The HA gene was similar to that of an H7N3 virus (ZJ12) from a nearby region (Zhejiang province) in China. All the internal gene segments were closely related to those from other avian H9N2 viruses.

The sources of avian influenza infection are mainly poultry or wild birds, which are carriers or sufferers of the disease. However, humans can be infected with avian influenza viruses via several transmission routes such as through direct avian-to-human contact, which is the most common mode of transmission; environment-to-human transmission; and mother-to-child vertical transmission [16-19]. Our study showed that 6 cases of pneumonia caused by H7N9 were detected in the area with close proximity to the Fifth People’s Hospital of Shanghai within a 2 week-period from 19 February and 5 March 2013. Among the 6 patients, 2 were related (father and son); in addition, several days prior to disease onset, the younger son (age, 55 years) developed high fever, cough, dyspnea, and rapidly developed ARDS, which was similar to the disease course of the other 2 family members who had confirmed cases of H7N9 infection. However, the H7N9 virus was not detected in his blood and respiratory specimens. 

The 6 patients lived in the proximity of the 2 markets where live birds were traded, and 2 patients had a history of exposure to birds; 1 had a history of suspected exposure to birds, and 1 patient worked in one of the markets. After the outbreak, the health department identified H7N9 viruses in carrier poultry in the 2 markets. Live bird trading was then forbidden. Thereafter, no additional cases of H7N9 infection were reported in the Minhang District. The above evidence suggests that the outbreak of infection caused by H7N9 viruses in the district was likely because of live bird carriers of H7N9 viruses in the 2 markets. The patients of this study had close contact with 122 people, who were mainly family members, relatives, and medical staff. These individuals were kept under medical observation for 1 week, and similar signs and symptoms to those of the patients were not observed. The lack of clinical infection signs in the contact individuals suggests that the efficiency of human-to-human transmission is low. Even without appropriate quarantine measures, the possibility of hospital infection is small. Therefore, a similar exposure history among direct relatives is believed to be the cause of clustering cases in a family. 

A general lack of immunity to avian influenza viruses leads to human infection, which can result in severe outcomes. Previously detected LPAI H7 avian influenza viruses were reported to cause mild symptoms, mainly conjunctivitis or flu-like symptoms, in humans [1,6,20,21], although H7N7 avian influenza viruses have been reported to also cause more serious complications such as pneumonia and ARDS [21,22]. For example, in the 2003 outbreak of the H7N7 virus in the Netherlands, 82 of the 89 cases (92.1%) manifested as conjunctivitis, whereas the remaining patients presented with an influenza-like illness [5]. HPAI H5N1 avian influenza viruses have caused more severe illnesses, from asymptomatic infection to fatal pneumonia and multiple organ failure than the H7 subtype. The characteristics of H5N1 avian influenza virus infection include a high case rate, high fatality rate, and development of shock in the early stages of disease [23,24]. The 6 patients infected with H7N9 viruses in this report showed signs and symptoms similar to those of patients infected with H5N1 viruses; all infected patients had pneumonia, with most rapidly progressing to ARDS. In the initial stage of disease, patients presented with fever and bloody sputum accompanied by chills, chest pain, nausea, and vomiting in some of the patients. Dyspnea developed 3–7 days after disease onset. Upper respiratory tract infection symptoms such as nasal congestion, runny nose, and sore throat, were not observed in any case, which was probably because of the lack of early diagnosis in patients with mild symptoms. Laboratory testing in the early stage showed that the WBC count was normal or lower than normal; the lymphocyte and platelet counts were low; and serum CRP, procalcitonin, CK, and LDH levels were elevated. Blood gas analysis also suggested hypoxemia and different degrees of excessive ventilation. Chest imaging suggested that consolidation and multiple ground-glass opacities of the lung developed in the early stage of the disease, and rapidly extended bilaterally. Patients with severe disease also showed ARDS-like symptoms. Furthermore, minor pleural effusion as well as subcutaneous and mediastinal emphysema were observed in some patients with severe disease. The above evidence suggests that fever, cough, hemoptysis, normal or low WBC levels, low lymphocyte counts, elevated CK and LDH levels, and pulmonary exudative lesions are significant characteristics of H7N9 infection.

Within 7 days of disease onset, H7N9 infection was confirmed in 4 of the 6 cases by RT-PCR assay and viral isolation using respiratory samples. The remaining 2 cases were confirmed using a specific antibody to the H7N9 virus in double serum samples from the acute and recovery stages. The results suggest that human infection with H7N9 viruses can be diagnosed early by RT-PCR of respiratory samples such as throat-swabs and sputum specimens, and by viral isolation from these samples. After admission, all patients were treated with broad-spectrum antibiotics, which were not effective in resolving symptoms. On days 3–8, all patients were treated with oral oseltamivir, and severely ill patients were treated with intravenous glucocorticoid, immunoglobulin, and thymosin. Several complications were observed, including ARDS, secondary bacterial infection, pleural effusion, left heart functional failure, neuropsychiatric symptoms, and rhabdomyolysis. ARDS developed on days 3–9 (mean, 6 days), the time course of which is consistent with that of reported cases of H5N1 infection in Thailand [25].

Factors affecting the prognosis of H7N9 viruses are unknown. Case 2 was a 69-year-old man with a history of hypertension and no history of drinking, and he recovered from the infection. Case 5 was a 41-year-old man with a history of smoking (smoking index, 4 pack-years) and no history of drinking or underlying disease, who also recovered. The ages of the other 4 patients were 87, 74, 27, and 63 years, and each had at least 1 underlying disease and a history of smoking and drinking; these patients ultimately died of the infection. These findings suggest that age may not be related to prognosis; however, underlying disease does appear to impact prognosis. The patients who recovered had no dyspnea, whereas those who died developed different degrees of dyspnea. In terms of laboratory testing, compared to the patients who died, the patients who recovered had only slightly varying platelet counts and CK and PO_2_ levels. In terms of complications, 1 patient who recovered had no complications, whereas the other developed secondary bacterial infections. In contrast, patients who died had 2–4 complications each. The time to treatment with oseltamivir therapy in the recovered cases was days 7 and 8, whereas it was days 3–8 in the patients who died. The above evidence implies that smoking, drinking, and underlying diseases may be related to the prognosis of H7N9 infection; dyspnea, decreased platelet counts, elevated CK levels, and hypoxemia may be associated with poor prognosis. The time to initiation of antiviral therapy seems to have no impact on prognosis, whereas the presence of complications may be associated with poor prognosis [10].

In conclusion, the first ever patient infected with H7N9 was treated at the Fifth People’s Hospital of Shanghai in February 2013. Within a 2-week period, several other cases of H7N9 infection emerged around 2 markets near the hospital. Fever, cough, sputum with blood, low lymphocyte counts, elevated CK and LDH levels, and pulmonary exudative lesions are significant characteristics of H7N9 infection, which easily progresses to ARDS. Among the cases, there was family clustering, which led to a high suspicion of contagious respiratory virus infection. In the early stage, human infection with H7N9 can be diagnosed by RT-PCR and viral isolation from respiratory specimens. Smoking, drinking, underlying diseases, dyspnea, low platelet counts, elevated CK levels, hypoxemia, and complications may be related to poor prognosis. However, diagnosis and treatment may be delayed because of the limited experience with this infection and small number of cases, which were among the first cases of H7N9 infection to be identified. Future studies are needed to elucidate the pathogenicity, transmissibility, and clinical features of H7N9 infection. In addition, techniques for early diagnosis to enable early administration of antiviral therapy and determination of the the factors affecting prognosis require further investigation.
